# Disinfection of the Mouth

**Published:** 1898-03

**Authors:** 


### Disinfection of the Mouth.

Montefusco's experiments (*Giornace internazionale della scienze Mediche*, fasc. 4 e 5) show that simply cleansing the buccal cavity with a brush sterilized by boiling and washing with sterilized water, continued for fifteen minutes, suffice to free the mouth from micro-organisms. Boric acid, benzoate of soda and chlorate of potash were found almost without action. The etheral oils, however (primarily, oil of cloves), proved most efficacious. The author deems mouth-washings with the essential oils of special prophylactic value in diphtheria and pneumonia.

